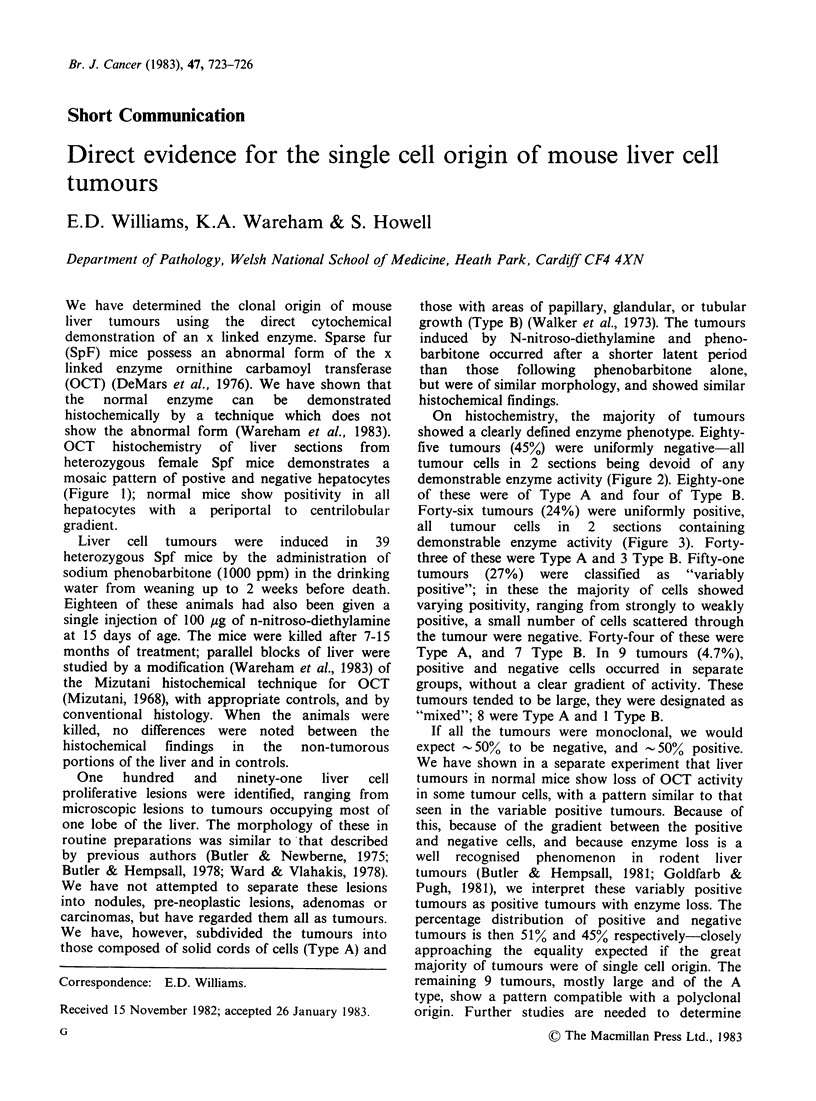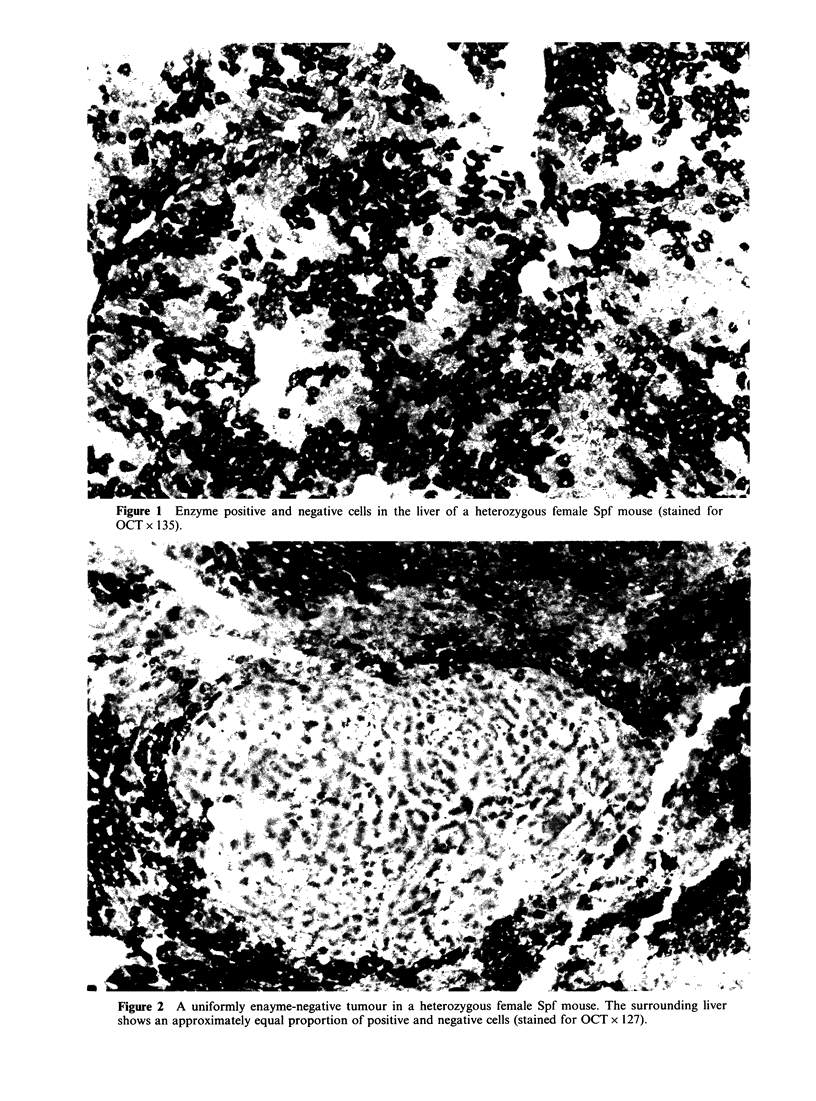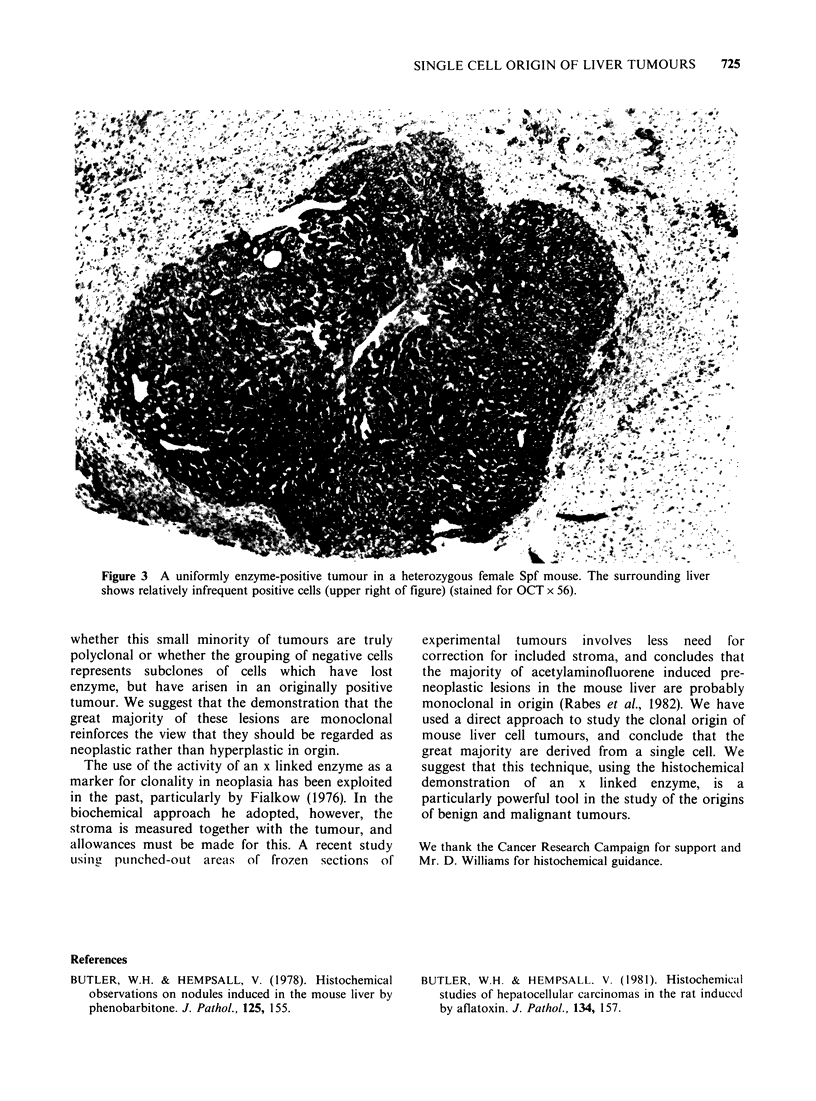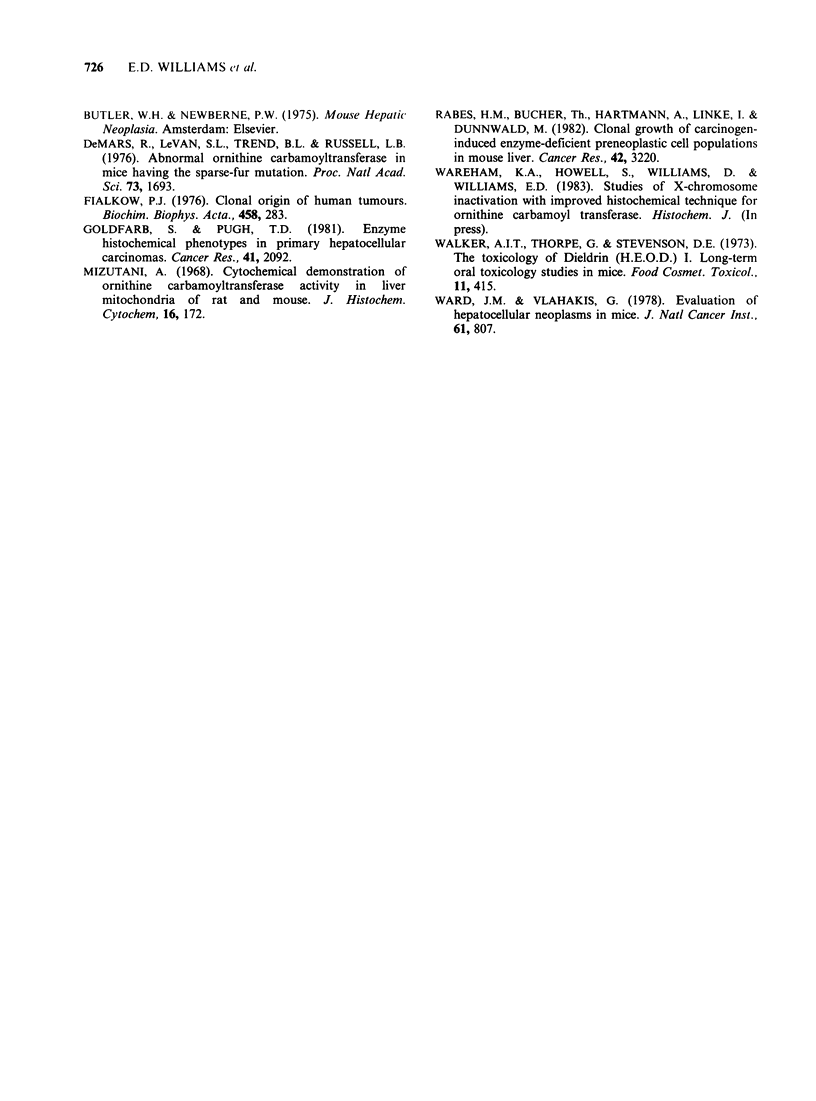# Direct evidence for the single cell origin of mouse liver cell tumours.

**DOI:** 10.1038/bjc.1983.112

**Published:** 1983-05

**Authors:** E. D. Williams, K. A. Wareham, S. Howell

## Abstract

**Images:**


Br. J. Cancer (1983), 47, 723–726

### Short Communication

### Direct evidence for the single cell origin of mouse liver cell tumours

E.D. Williams, K.A. Wareham & S. Howell

*Department of Pathology, Welsh National School of Medicine, Heath Park, Cardiff CF4 4XN*

We have determined the clonal origin of mouse liver tumours using the direct cytochemical demonstration of an x linked enzyme. Sparse fur (SpF) mice possess an abnormal form of the x linked enzyme ornithine carbamoyl transferase (OCT) (DeMars *et al.*, 1976). We have shown that the normal enzyme can be demonstrated histochemically by a technique which does not show the abnormal form (Wareham *et al.*, 1983). OCT histochemistry of liver sections from heterozygous female Spf mice demonstrates a mosaic pattern of postive and negative hepatocytes (Figure 1); normal mice show positivity in all hepatocytes with a periportal to centrilobular gradient.

Liver cell tumours were induced in 39 heterozygous Spf mice by the administration of sodium phenobarbitone (1000 ppm) in the drinking water from weaning up to 2 weeks before death. Eighteen of these animals had also been given a single injection of 100 $\mu$g of n-nitroso-diethylamine at 15 days of age. The mice were killed after 7-15 months of treatment; parallel blocks of liver were studied by a modification (Wareham *et al.*, 1983) of the Mizutani histochemical technique for OCT (Mizutani, 1968), with appropriate controls, and by conventional histology. When the animals were killed, no differences were noted between the histochemical findings in the non-tumorous portions of the liver and in controls.

One hundred and ninety-one liver cell proliferative lesions were identified, ranging from microscopic lesions to tumours occupying most of one lobe of the liver. The morphology of these in routine preparations was similar to that described by previous authors (Butler & Newberne, 1975; Butler & Hempsall, 1978; Ward & Vlahakis, 1978). We have not attempted to separate these lesions into nodules, pre-neoplastic lesions, adenomas or carcinomas, but have regarded them all as tumours. We have, however, subdivided the tumours into those composed of solid cords of cells (Type A) and those with areas of papillary, glandular, or tubular growth (Type B) (Walker *et al.*, 1973). The tumours induced by N-nitroso-diethylamine and phenobarbitone occurred after a shorter latent period than those following phenobarbitone alone, but were of similar morphology, and showed similar histochemical findings.

On histochemistry, the majority of tumours showed a clearly defined enzyme phenotype. Eighty-five tumours (45%) were uniformly negative—all tumour cells in 2 sections being devoid of any demonstrable enzyme activity (Figure 2). Eighty-one of these were of Type A and four of Type B. Forty-six tumours (24%) were uniformly positive, all tumour cells in 2 sections containing demonstrable enzyme activity (Figure 3). Forty-three of these were Type A and 3 Type B. Fifty-one tumours (27%) were classified as "variably positive"; in these the majority of cells showed varying positivity, ranging from strongly to weakly positive, a small number of cells scattered through the tumour were negative. Forty-four of these were Type A, and 7 Type B. In 9 tumours (4.7%), positive and negative cells occurred in separate groups, without a clear gradient of activity. These tumours tended to be large, they were designated as "mixed"; 8 were Type A and 1 Type B.

If all the tumours were monoclonal, we would expect ~50% to be negative, and ~50% positive. We have shown in a separate experiment that liver tumours in normal mice show loss of OCT activity in some tumour cells, with a pattern similar to that seen in the variable positive tumours. Because of this, because of the gradient between the positive and negative cells, and because enzyme loss is a well recognised phenomenon in rodent liver tumours (Butler & Hempsall, 1981; Goldfarb & Pugh, 1981), we interpret these variably positive tumours as positive tumours with enzyme loss. The percentage distribution of positive and negative tumours is then 51% and 45% respectively—closely approaching the equality expected if the great majority of tumours were of single cell origin. The remaining 9 tumours, mostly large and of the A type, show a pattern compatible with a polyclonal origin. Further studies are needed to determine

Correspondence: E.D. Williams.

Received 15 November 1982; accepted 26 January 1983.

© The Macmillan Press Ltd., 1983

**Figure 1** Enzyme positive and negative cells in the liver of a heterozygous female Spf mouse (stained for OCT × 135).

**Figure 2** A uniformly enayme-negative tumour in a heterozygous female Spf mouse. The surrounding liver shows an approximately equal proportion of positive and negative cells (stained for OCT × 127).

**Figure 3** A uniformly enzyme-positive tumour in a heterozygous female Spf mouse. The surrounding liver shows relatively infrequent positive cells (upper right of figure) (stained for OCT x 56).

whether this small minority of tumours are truly polyclonal or whether the grouping of negative cells represents subclones of cells which have lost enzyme, but have arisen in an originally positive tumour. We suggest that the demonstration that the great majority of these lesions are monoclonal reinforces the view that they should be regarded as neoplastic rather than hyperplastic in orgin.

The use of the activity of an x linked enzyme as a marker for clonality in neoplasia has been exploited in the past, particularly by Fialkow (1976). In the biochemical approach he adopted, however, the stroma is measured together with the tumour, and allowances must be made for this. A recent study using punched-out areas of frozen sections of experimental tumours involves less need for correction for included stroma, and concludes that the majority of acetylaminofluorene induced pre-neoplastic lesions in the mouse liver are probably monoclonal in origin (Rabes *et al.*, 1982). We have used a direct approach to study the clonal origin of mouse liver cell tumours, and conclude that the great majority are derived from a single cell. We suggest that this technique, using the histochemical demonstration of an x linked enzyme, is a particularly powerful tool in the study of the origins of benign and malignant tumours.

We thank the Cancer Research Campaign for support and Mr. D. Williams for histochemical guidance.

**References**

BUTLER, W.H. & HEMPSALL, V. (1978). Histochemical observations on nodules induced in the mouse liver by phenobarbitone. *J. Pathol.*, **125**, 155.

BUTLER, W.H. & HEMPSALL, V. (1981). Histochemical studies of hepatocellular carcinomas in the rat induced by aflatoxin. *J. Pathol.*, **134**, 157.

BUTLER, W.H. & NEWBERNE, P.W. (1975). *Mouse Hepatic Neoplasia*. Amsterdam: Elsevier.

DeMARS, R., LeVAN, S.L., TREND, B.L. & RUSSELL, L.B. (1976). Abnormal ornithine carbamoyltransferase in mice having the sparse-fur mutation. *Proc. Natl Acad. Sci.* **73,** 1693.

FIALKOW, P.J. (1976). Clonal origin of human tumours. *Biochim. Biophys. Acta.,* **458,** 283.

GOLDFARB, S. & PUGH, T.D. (1981). Enzyme histochemical phenotypes in primary hepatocellular carcinomas. *Cancer Res.,* **41,** 2092.

MIZUTANI, A. (1968). Cytochemical demonstration of ornithine carbamoyltransferase activity in liver mitochondria of rat and mouse. *J. Histochem. Cytochem,* **16,** 172.

RABES, H.M., BUCHER, Th., HARTMANN, A., LINKE, I. & DUNNWALD, M. (1982). Clonal growth of carcinogen-induced enzyme-deficient preneoplastic cell populations in mouse liver. *Cancer Res.,* **42,** 3220.

WAREHAM, K.A., HOWELL, S., WILLIAMS, D. & WILLIAMS, E.D. (1983). Studies of X-chromosome inactivation with improved histochemical technique for ornithine carbamoyl transferase. *Histochem. J.* (In press).

WALKER, A.I.T., THORPE, G. & STEVENSON, D.E. (1973). The toxicology of Dieldrin (H.E.O.D.) I. Long-term oral toxicology studies in mice. *Food Cosmet. Toxicol.,* **11,** 415.

WARD, J.M. & VLAHAKIS, G. (1978). Evaluation of hepatocellular neoplasms in mice. *J. Natl Cancer Inst.,* **61,** 807.